# Tobacco Use Prevention and Cessation Programme For Pregnant Women Accessing Antenatal Care In Urban Public Health Facilities In Southern India

**DOI:** 10.1093/eurpub/ckac129.258

**Published:** 2022-10-25

**Authors:** S Thomas, H Lee

**Affiliations:** 1 Public Health Foundation of India, The Ramalingaswami Centre on Equity, Bengaluru, India; 2 Oral Health Workgroup, WFPHA, Geneva, Switzerland

## Abstract

**Backgrounds and problem:**

By 2030, tobacco use is estimated to kill more than 8 million people worldwide annually, with LMICs accounting for over 80% of those deaths. In India, about 4.6% of women continue to use tobacco mainly (> 80%) in smokeless (SLT) form during pregnancy. This may lead to: higher risk of anemia (∼ 70%), hypertension, and postpartum hemorrhage; poor fetal development; and 2-3 times higher rate of low-birth and stillbirth babies.

**Importance:**

Anti-tobacco initiatives often focus on smoking over SLT, which is commonly consumed by women. Drivers of SLT use among women include: cultural appropriateness, medicinal benefits, and poor social determinants of health. These factors must be taken into account while formulating effective anti-tobacco interventions for pregnant women, ensuring safe motherhood and neonatal health.

**Solution:**

We propose integrating oral health interventions in mitigating tobacco use within the existing antenatal care (ANC) model. Women enrolled in public health facilities in an urban poor neighborhood in South India receive oral health education (OHE) integrated into ANC. The intervention promotes oral hygiene habits, dietary advices, improving dental healthcare utilization, and sensitization on the ill-effects of tobacco-use. Training for ANC providers focuses on delivering antenatal tobacco screening, cessation, and referral services. This includes expanding the medical history to record tobacco use, conduct oral examination and referrals to dentists and/or tobacco cessation centres. Final outcomes of OHE will be measured using pre and post KAP surveys informed by WHO Oral Health Surveys; and training programme using in-depth interviews among providers. Desired policy change is the inclusion of tobacco prevention and cessation programme in the Indian ANC guidelines.

